# Gulf War Illness: C-Reactive Protein is Associated with Reduction of the Volume of Hippocampus and Decreased Fractional Anisotropy of the Fornix

**DOI:** 10.29245/2572.942x/2020/3.1272

**Published:** 2020-08-11

**Authors:** Peka Christova, Lisa M. James, Adam F. Carpenter, Scott M. Lewis, Rachel A. Johnson, Brian E. Engdahl, Apostolos P. Georgopoulos

**Affiliations:** 1 Brain Sciences Center, Department of Veterans Affairs Health Care System, Minneapolis, MN, 55417, USA; 2 Department of Neuroscience, University of Minnesota Medical School, Minneapolis, MN 55455, USA; 3 Department of Psychiatry, University of Minnesota Medical School, Minneapolis, MN 55455, US; 4 Department of Neurology, University of Minnesota Medical School, Minneapolis, MN 55455, USA; 5 Department of Psychology, University of Minnesota Medical School, Minneapolis, MN 55455, USA

**Keywords:** Gulf War Illness, C-reactive protein, Hippocampus, Fornix, Fractional Anisotropy

## Abstract

Memory and mood impairments are among the most commonly reported symptoms in veterans with Gulf War Illness (GWI), suggesting hippocampal involvement. Several studies have also documented evidence of inflammation in GWI. The aim of the present study was to evaluate the association between C-reactive protein (CRP), a marker of inflammation, and hippocampal volume and microstructural alterations of its major output, the fornix. Sixty-three veterans with GWI provided blood samples for evaluation of CRP and underwent a 3T magnetic resonance imaging scan from which hippocampal volume and fornix fractional anisotropy (FA) were obtained. Results demonstrated that CRP was significantly and negatively associated with hippocampal volume and fornix FA in GWI. Given the known closely interwoven associations between inflammation and neurodegeneration, it is possible that the effects we observed could be due to neurodegeneration, secondary to chronic neuroinflammation. Finally, given the known association of hippocampus to memory and mood disorders, our findings provide new insights into memory and mood alterations associated with GWI.

## Introduction

Nearly thirty years after the 1990–1991 Persian Gulf War, Gulf War veterans continue to report a variety of chronic, often disabling symptoms, commonly referred to as Gulf War Illness (GWI)^1,2^. The cause of GWI has not been conclusively identified; however, mounting evidence suggests GWI is best characterized as a neuroimmune disorder^3,4^, with several studies documenting various structural and functional brain abnormalities^5–7^ and evidence of elevated inflammatory markers such as C-reactive protein (CRP) in veterans with GWI^8–11^. Mood and memory impairments are particularly prominent among veterans with GWI^2^, suggesting hippocampal involvement; however, the effect of CRP on hippocampal alterations in GWI has not previously been investigated.

Several hippocampal abnormalities have been associated with GWI including reductions in total hippocampal and/or hippocampal subfield volume in Gulf War veterans exposed to organophosphates^12–15^, alterations in hippocampal cerebral blood flow following cholinergic challenge^16^, and reduced hippocampal N-acetyl apartate/creatinine ratio, indicative of neuronal and/or axonal loss or dysfunction^17^. Research using animal models of GWI have reported similar hippocampal alterations^18–20^, and have provided evidence that systemic and hippocampal inflammation underlie memory and mood impairment associated with GWI^21^, highlighting the likely influence of systemic inflammation on hippocampal macro- and microstructure.

The use of diffusion weighted imaging (DWI), has contributed to fundamental advances in human brain anatomy by providing a window to white matter microstructure^22,23^. DWI measures water molecule diffusion along different directions including in axons where water is constrained (by the myelin sheath) to diffuse mainly along the long axis of the axon. Basser and colleagues^24,25^ mathematically described the diffusion processes by a diffusion tensor, starting the field of a diffusion tensor imaging (DTI). DTI is the widely used for studying white matter changes in the healthy brain across the lifespan^26–29^, as well as in conditions affecting the brain^30–35^.

With regard to GWI, research using DTI has been limited and findings have been inconsistent. For example, Rayhan and colleagues suggested that increased axial diffusivity in the right inferior fronto-occipital fasciculus may be a biomarker of GWI^36^; however, that finding was not replicated in a subsequent study that reported increased axial diffusivity elsewhere^37^. A study of hippocampal gray matter microstructure in GW veterans with and without suspected in-theater exposure to organophosphates found that mean diffusivity predicted suspected in-theater organophosphate exposure^15^. The authors did not find evidence of differences in fractional anisotropy (FA) or gray matter density between the two exposure groups though they noted that FA is better suited to study microstructural changes in white matter than in gray matter.

One of the major white matter outputs of the hippocampus is the fornix, a bundle of fibers that connects the hippocampus to subcortical structures in the basal forebrain and diencephalon. This hippocampal-fornix circuitry is a part of the Papez circuit^38^. The fornix and hippocampus affect each other such that the damage to the integrity of the fornix causes disruption of hippocampal function and vice versa^39–41^. Hippocampal functional and structural abnormalities have been shown to be associated with fornix white matter integrity disruption in various conditions shown to be associated with fornix white matter disruption in various conditions, including conditions with prominent CNS inflammation such as multiple sclerosis and Alzheimer’s disease^41–45^.

Systemic inflammation influences blood-brain barrier (BBB) permeability^46^, and the hippocampus is particularly sensitive to BBB breakdown^47^. GWI has been associated with inflammation^8–11^, and, separately, inflammation has been linked to reduced hippocampal volume in GW veterans with posttraumatic stress disorder^48^. Finally, CRP is associated with cerebral microstructural integrity alterations as evidenced by reduced FA^49^. FA shows the degree of anisotropy and restriction of water diffusion by macromolecules and cell membranes. It varies in magnitude ranging from 0 to 1, depending on the characteristics of tissue microstructure^50^. Values close to 1 represent an almost unidirectional anisotropic diffusion, while lower values of FA can be the result of compromised white matter integrity.

## Materials and Methods

### Participants

Sixty-three GWI patients (59 men and 4 women) were studied after providing an informed consent in accordance with the Declaration of Helsinki. GWI status was determined using a self-report symptom checklist that permits classification as GWI case or control according to the Center for Disease Control^1^ and the Kansas criteria^2^. The Center for Disease Control definition requires one or more symptoms in at least two domains that include fatigue, pain, or mood and cognition. The more restrictive Kansas criteria requires that veterans report moderate to severe symptoms in at least 3 of 6 domains: fatigue, pain, neurological/cognitive/mood, skin, gastrointestinal, and respiratory. All GWI veterans in the present study met both case definitions. Consistent with the Kansas criteria case definition, veterans were excluded from the study if they reported medical or psychiatric conditions that could account for GWI symptoms or impair reporting2. Individuals with traumatic brain injury were also excluded from the study. The study was approved by the University of Minnesota and Minneapolis VA Health Care System Institutional Review Boards.

#### Body Mass Index (BMI)

BMI was computed using the height and weight of the participant (BMI = kg/m^2^).

#### CRP

Non-fasting peripheral venous blood samples were collected for evaluation of high sensitivity C- reactive protein and analyzed using standard procedures by the Minneapolis VAHCS Clinical Laboratory.

#### Magnetic Resonance Imaging (MRI) acquisition

All data were acquired using a Philips 3T MR scanner (Achieva, Philips Healthcare, Best, The Netherlands). In the initial phase of the study, data were acquired from 17 participants using a phased array SENSitivity Encoding (SENSE) 8-channel head coil for reception. For each participant a high resolution T1-weighted Turbo Field Echo (T1w TFE SENSE) was obtained (168 sagittal slices, TR = 8.1932 ms, TE =3.7520 ms, Acquisition matrix 240 × 240, Flip angel 8 deg., voxel size 0.9375 × 0.9375 × 1 mm). A T2-weighted image (T2w VISTA HR SENSE) was also obtained (180 slices, TR = 2500 ms, TE =363.072 ms, Acquisition matrix 252 × 252, voxel size =0.7813 ×0.7813 × 1 mm). Subsequently, upgrades were applied to the system and data were acquired from the remainder 46 participants using a phased array SENSitivity Encoding (SENSE) 15-channel head coil for reception. For each participant a high resolution T1-weighted Turbo Field Echo (T1w TFE SENSE) was obtained (168 sagittal slices, TR =8.0928 ms, TE = 3.698 ms, Acquisition matrix 240 × 240, Flip angel 8 deg., voxel size 0.7500 mm × 0.7500 mm × 1 mm). The T2-weighted (T2w VISTA HR SENSE) was also obtained (168 slices, TR = 2500 ms, TE = 370.346 ms, Acquisition matrix 240 × 240, voxel size = 0.7500 mm ×0.7500 mm × 1 mm).

Diffusion weighted images (DWI, DTI_medium_iso_E) consisted of a single-shot echo-planar imaging sequence (EPI, TR=11.023 s, TE = 55 ms, Acquisition matrix 112 × 112, 70 slices with 2 mm thickness without gap, in-plane resolution = 0.875 mm × 0.875 mm). Images were acquired in the axial plane with diffusion gradients applied in 15 non-collinear directions with a b-value of 1000 s/mm^2^ and one non-diffusion weighted image with a b-value of 0 s/mm^2^. In advance of each acquisition a capsule of Vitamin E was taped to the participant’s right temple to determine orientation in the imaged data.

## MRI Image Processing

### Structural MRI

A 704-core High Performance Computing system (CentOS 6.5 Linux, Rocks 6.1.1) with Matlab R2016 (64 bit), Human Connectome Project (HCP humanconnectome. org) pipeline with FreeSurfer (FS; http://surfer.nmr.mgh.harvard.edu) HCP version (freesurfer-hpc) was used for data processing, as we describe in detail elsewhere^7^. Briefly, we used a modified version of FS, implemented in the structural HCP pipeline, which utilizes both T1-w and T2-w images to eliminate uncertainty and improve pial surface reconstruction^51^. FS segments subcortical structures by using a volume-based analysis pipeline. In this analysis subcortical regions were automatically labeled^52,53^. The volumes of left and right hippocampi were averaged to obtain the average hippocampal volume that was used in subsequent analyses.

## DTI

Diffusion data were analyzed using the default parameter settings in the diffusion MR toolbox ExploreDTI, version 4.8.6 (www.exploredti.com^54^). Anatomical T1-w images for each participant were linearly transformed into Montreal Neurological Institute (MNI) 152 space using AFNI (Analysis of Functional NeuroImages, afni.nimh.nih.gov). Diffusion weighted images were corrected for head movement and eddy current induced geometric distortions using the procedure described elsewhere^54^ and corrected for EPI/susceptibility distortion^55^. Then the image was warped to an ‘undistorted’ T1-w modality, in the MNI-152 template using the ELASTIX approach^56^ with non-rigid registration. The above steps were applied to the data obtained from each participant and done in one interpolation to reduce blurring effects, resulting in streamlined files. The quality of each processing step was visually inspected. The validity of co-registrations was checked by overlaying the respective images for each participant.

ExploreDTI provides the Mori standard labeled atlas in the same MNI standard space (ICBM-DTI-81^57,58^). By warping the atlas template and transforming the associate labels to each individual data set, the mean values of diffusion metric fractional anisotropy (FA) of the body and column of the fornix (called “fornix” thereafter) was obtained for each participant. The following formula was used for calculating FA:

(1) $$\text{FA}=\sqrt{\frac{3}{2}}\frac{\sqrt{{\left(\lambda_{1}-\hat{\lambda }\right)}^{2}+{\left(\lambda_{2}-\hat{\lambda }\right)}^{2}+{\left(\lambda_{3}-\hat{\lambda }\right)}^{2}}}{\sqrt{\lambda_{1}^{2}+\lambda_{2}^{2}+\lambda_{3}^{2}}}\;\hat{\lambda }=\left(\lambda_{1}+\lambda_{2}+\lambda_{3}\right)/3$$

where is the mean of the eigenvalues of the diffusion tensor.

## Data analysis

Standard statistical methods were used to analyze the data, including descriptive statistics and linear regression. Regression coefficients were compared, as needed, using the following formula^59^:

(2) $$Z=\frac{b_{1}-b_{2}}{\sqrt{SE{b_{1}}^{2}+SE{b_{2}}^{2}}}$$

where z is the normal deviate, $b_{1}$ and $b_{2}$ denote regression coefficients to be compared, and SE are their standard errors. All statistical analyses were done using the IBM-SPSS statistical package (version 23).

## Results

### Distributions of data and descriptive statistics

#### Age.

The frequency distribution of age is shown in Figure 1; the mean ± SEM age was 55.2 ± 1.12 y.

#### BMI.

The mean ± SEM BMI was 31.3 ± 0.64 (N = 63).

#### CRP.

The frequency distribution of CRP values (mg/dl) is shown in the left panel of Figure 2 It can be seen that it is skewed to the right, deviating appreciably from a normal distribution, as evidenced by the probability-probability plot in right panel of Figure 2. A logarithmic transformation was applied to bring the distribution close to normal (Figure 3):

(2) $$\text{ln}\left(\text{CRP}\right)={\text{log}}_{\text{e}}\left(\text{CRP}\right)$$

A detailed analysis of this distribution showed the absence of outliers. The mean ± SEM of ln(CRP) was 0.674 ± 0.122 (N = 63).

#### Volume of left hippocampus.

The frequency distribution of the volume of the left hippocampus is shown in the left panel of Figure 4; the mean ± SEM was 4205.32 ± 60.76 mm^3^ (N = 63).

#### Volume of right hippocampus.

The frequency distribution of the volume of the right hippocampus is shown in the right panel of Figure 4; the mean ± SEM was 4284.30 ± 64.05 mm^3^ (N = 63).

#### Average volume of hippocampus.

The frequency distribution of the volume of the averaged left and right hippocampus was 4244.81 ± 61.05 mm^3^ (N = 63).

#### Fractional anisotropy of the fornix.

The frequency distribution of the FA of the fornix is shown in Figure 5; the mean ± SEM was 0.433 ± 0.012.

### Associations

#### Effect of total intracranial volume (eTIV).

eTIV did not have a significant effect on the volumes of left, right, and average hippocampus (P > 0.1 for all; linear regression); therefore, eTIV was not used in subsequent analyses.

#### Effect of gender.

Gender did not have a significant effect on the volumes of left, right, and average hippocampus, and the FA of fornix (P > 0.5 for all); therefore, gender was not used in subsequent analyses.

#### Effect of age.

Age had a significant negative effect on the volumes of left, right, and average hippocampus, and the FA of fornix. The regression equations were as follows.


(3) $$\text{Left}\;\text{hippocampal}\;\text{volume}\;(mm^{3})=5374.71-20.92\;y\;[P=0.002;\;\text{Figure}\;6,\;\text{left}\;\text{panel}]$$

(4) $$\text{Right}\;\text{hippocampal}\;\text{volume}\;\left({mm}^{3}\right)=5337.99-20.64\;y\;[P=0.004;\;\text{Figure}\;6,\;\text{right}\;\text{panel}]$$

(5) $$\text{Average}\;\text{hippocampal}\;\text{volume}\;\left({mm}^{3}\right)=5406.35-20.78\;y\;[P=0.002;\;\text{Figure}\;7]$$

(6) FornixFA=0.761-0.00586y[P=0.00001;Figure8]

Since age had a significant effect, it was added as a covariate in all subsequent analyses below. Finally, age and ln(CRP) were not significantly correlated (r = −0.146, P = 0.254).

#### Effect of BMI.

BMI did not have a significant effect on the volumes of left, right, and average hippocampus, and the FA of fornix (P > 0.3 for all); therefore, BMI was not used in subsequent analyses.

#### Effect of CRP.

CRP had a negative effect on the volumes of left, right, and average hippocampus, and the FA of fornix. This effect did not reach statistical significance for the left hippocampal volume but was significant for the other measures. The regression equations were as follows.


(7) $$\text{Left}\;\text{hippocampal}\;\text{volume}\;(mm^{3})=5546.72-22.68\;y-109.76\;\text{ln}\;(\text{CRP})$$

[P for ln(CRP) = 0.065; Figure 9, left panel]

(8) $$\text{Right}\;\text{hippocampal}\;\text{volume}\;(mm^{3})=5658.18-22.89\;y-140.51\;\text{ln}\;(\text{CRP})$$

[P for ln(CRP) = 0.025; Figure 9, right panel]

(9) $$\text{Average}\;\text{hippocampal}\;\text{volume}\;(mm^{3})=5602.45-22.78\;y-125.14\;\text{ln}\;(\text{CRP})$$

[P for ln(CRP) = 0.025; Figure 10]

(10) FornixFA=0.808-0.00634y-0.03ln(CRP)

[P for ln(CRP) = 0.006; Figure 11]

## Comparison of regression coefficients for age

A regression coefficient (slope, beta) indicates the rate by which the dependent variable changes per unit change of the independent variable. When estimated under different conditions, a comparison of coefficients between conditions could provide information regarding the possible effect of the condition on the coefficient. Here we compared the coefficients for age between the left and right hippocampal volumes to find out whether the effect of age differed significantly between the left and right hippocampi. The regression coefficients for age (and their standard errors in parentheses) were −20.92 (6.45) and −20.64 (6.87) for the left and right hippocampus, respectively. Applying the formula of Equation 2 above, we get z = 0.03, indicating that the two regression coefficients do not differ significantly. Therefore, age affects the volumes of the left and right hippocampus in the same way.

We also compared the regression coefficients for age obtained in the absence or presence of ln(CRP) as an additional covariate. A significant difference of the coefficients between the two cases would indicate a moderating effect of ln(CRP) on the age effect. In the absence of ln(CRP) as a covariate, the coefficients for age (and their standard errors in parentheses) were −20.92 (6.45), −20.64 (6.87), and −0.00586 (0.00122) for the left and right hippocampal volumes and FA of the fornix, respectively. In the presence of ln(CRP), those coefficients were −22.68 (6.39), −22.89 (6.71), and −0.00634 (0.001167) for the left and right hippocampal volumes and FA of the fornix, respectively. Using the formula of Equation 2 to compare corresponding coefficients, we get z = 0.193, z = 0.234, and z = 0.286 for left and right hippocampal volumes and fornix FA, respectively. These results indicate that the inclusion of ln(CRP) as a covariate did not alter the effect of age on hippocampal volumes and fornix FA.

## Discussion

In this study, we examined the effect of inflammation, as assessed by CRP, on fornix microstructural integrity and gray matter hippocampal volume in GWI veterans. The findings demonstrated that higher CRP levels are associated with greater white matter fornix alterations and reduced hippocampal gray matter volume. The results further highlight the role of inflammation in GWI and document specific effects of inflammation on the hippocampus and fornix in GWI.

While the brain was historically thought to be immune-privileged, it is now widely recognized that the immune system and brain interact and profoundly influence each other^60^. Under healthy conditions, the brain’s resident immune cells, microglia, influence learning, memory, neural plasticity, and neurogenesis; however, chronic immune system activation impairs these processes and contributes to mood alterations^61–63^. GWI commonly involves memory and mood impairments^2,^ and is increasingly viewed as a neuroimmune condition^3,4^. Several studies have documented inflammation in GWI^8–11^ with mounting evidence suggesting that pathogen exposure in those lacking immunogenetic protection contributes to GWI and associated inflammation^64–70^. Here we documented that peripheral inflammatory signaling is associated with white matter abnormalities and reduced gray matter volume in GWI. It has been shown that low-grade systemic inflammation alone may be enough to lead to neurological dysfunction^71^. Systemic inflammation may also directly contribute to microcirculatory dysfunction and cerebrovascular permeability^72^ and increased pro-inflammatory signaling within the central nervous system^73^.

Inflammation affects the brain by producing pro-inflammatory cytokines such as interleukin 6 (IL-6), interleukin-1β (IL-1β), tumor necrosis factor-α (TNFα), and reactive oxygen species^61,74^. Pro-inflammatory cytokines are measured in the serum in the periphery, but they are found as mediators of many central nervous system effects^75^ and have been shown to increase with age, stress, and fatigue^76^. Notably, a recent study found no elevation of peripheral pro-inflammatory cytokines in GWI compared to healthy controls despite evidence of neuroinflammation^77^. Others have also failed to find evidence of increased cytokines in GWI but have reported elevated CRP^9,10^, which had historically been thought to result from increased IL-6. Reevaluation of the causality between the inflammatory cytokines such as IL-6 and CRP found that they are interrelated and that they play both pro- and anti-inflammatory roles^78^, and that each can influence the other^74^. Using a more sensitive cytokine immunoassay, a recent study found increased IL-6 and CRP in veterans with GWI^79^.

Here we found CRP levels to be significantly and negatively correlated with fornix FA values and hippocampal volume, highlighting the influence of inflammation on both fornix microstructural integrity and hippocampal macrostructure. Notably, fornix degeneration has been shown to be correlated with hippocampal volume reductions across various conditions^43,45, 80–82^, though the direction of the effects is unclear. For healthy adults the integrity of the fornix has been shown to predict hippocampal volume, but only for the oldest age group^44^. A longitudinal study of individuals at-risk for Alzheimer’s disease found that baseline hippocampal volume predicted fornix microstructure 2 years later but the reverse – FA predicting hippocampal volume - was not supported^42^. The current study documented significant negative associations between an inflammatory marker (CRP) and hippocampal measures of volume and white matter integrity. Given the known closely interwoven associations between inflammation and neurodegeneration^83^, it is possible that the effects we observed could be due to neurodegeneration, secondary to chronic neuroinflammation. Furthermore, because FA is a fairly non-specific indicator of microstructural architecture^84^, the exact nature of the microstructural alterations in GWI remain to be elucidated.

## Limitations of the study

A limitation of the study concerns the possible partial volume effect of CSF contamination that may have affected the FA measurement of the fornix. In addition, our study did not permit differentiation of inflammation and neurodegeneration, especially given their intricate interrelations^83^.

## Figures and Tables

**Figurere 1.**
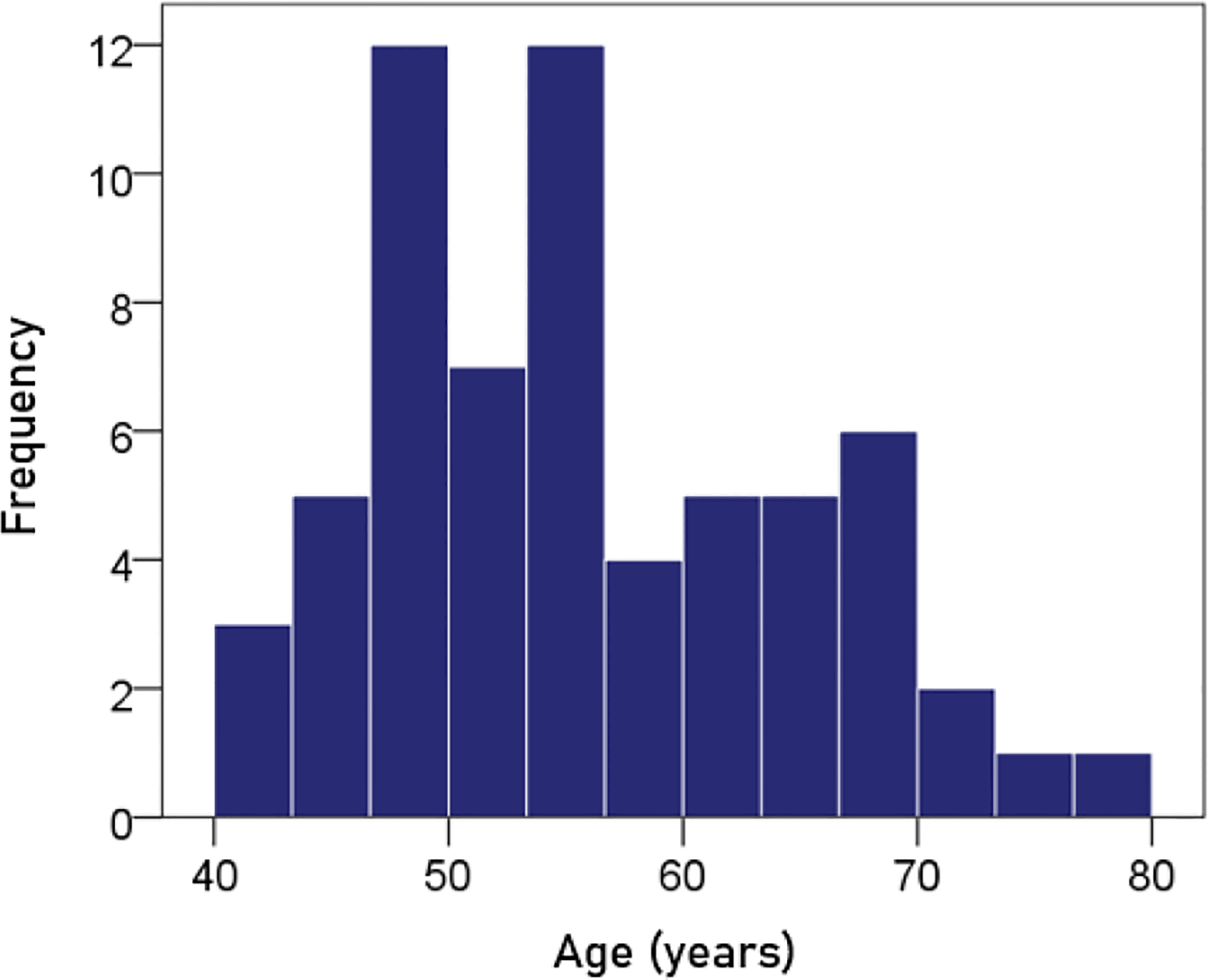
Frequency distribution of age. N = 63.

**Figurere 2.**
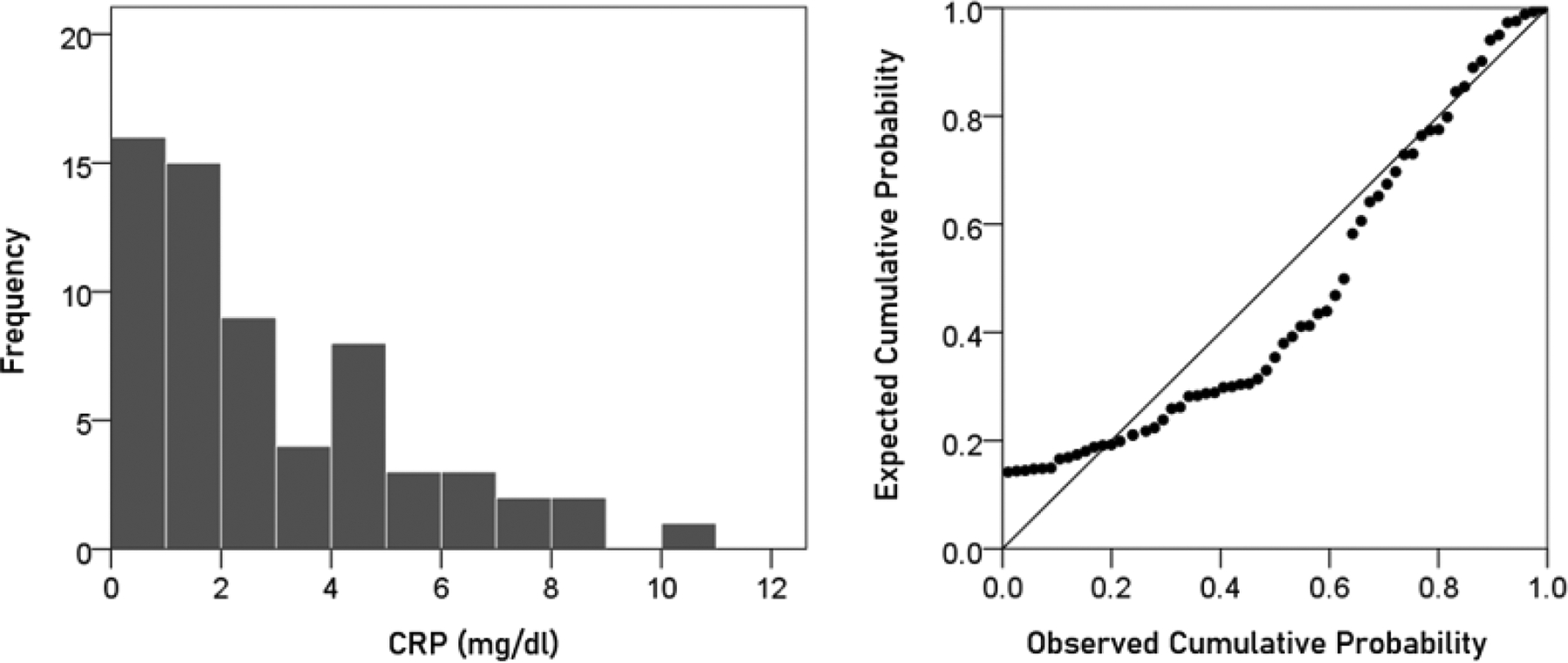
Frequency distribution of CRP (left panel) and corresponding probability-probability plot to indicate appreciable deviation from a normal distribution.

**Figurere 3.**
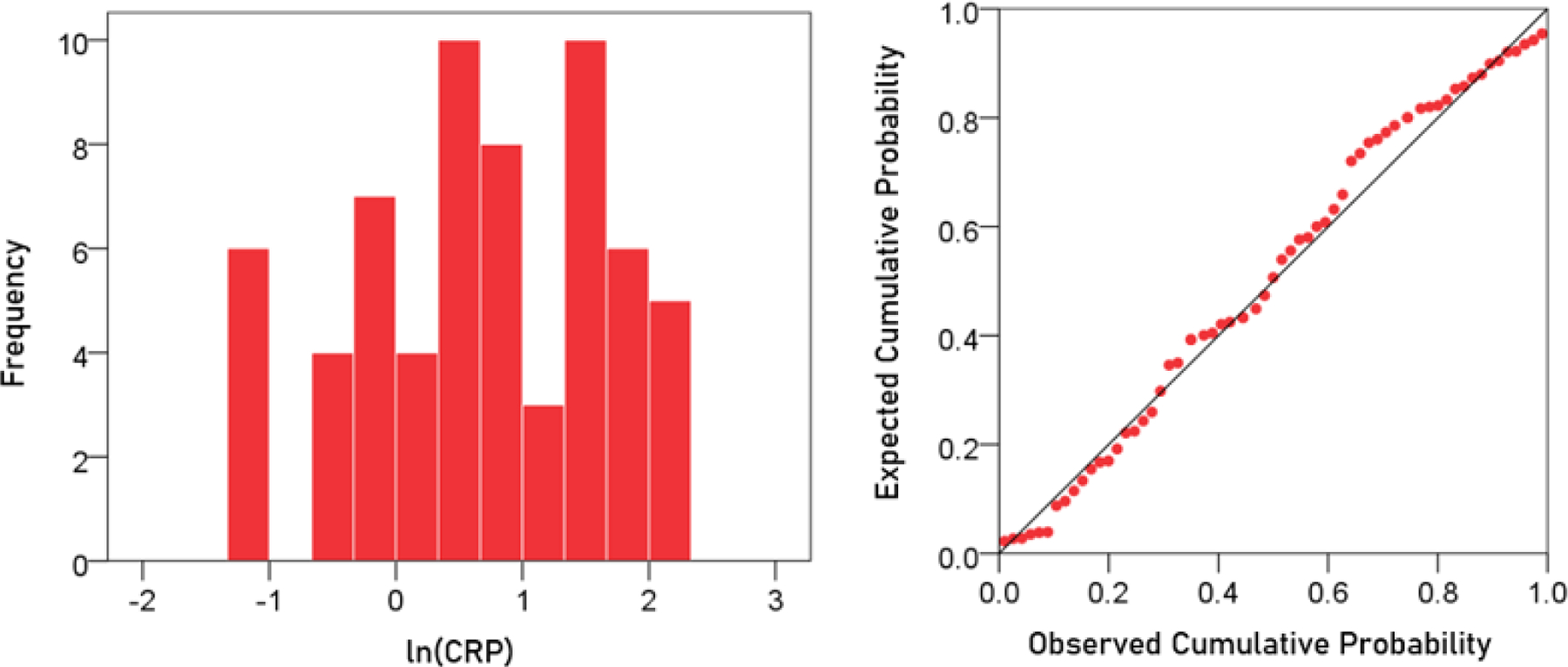
Frequency distribution of ln(CRP) (left panel) and corresponding probability-probability plot.

**Figurere 4.**
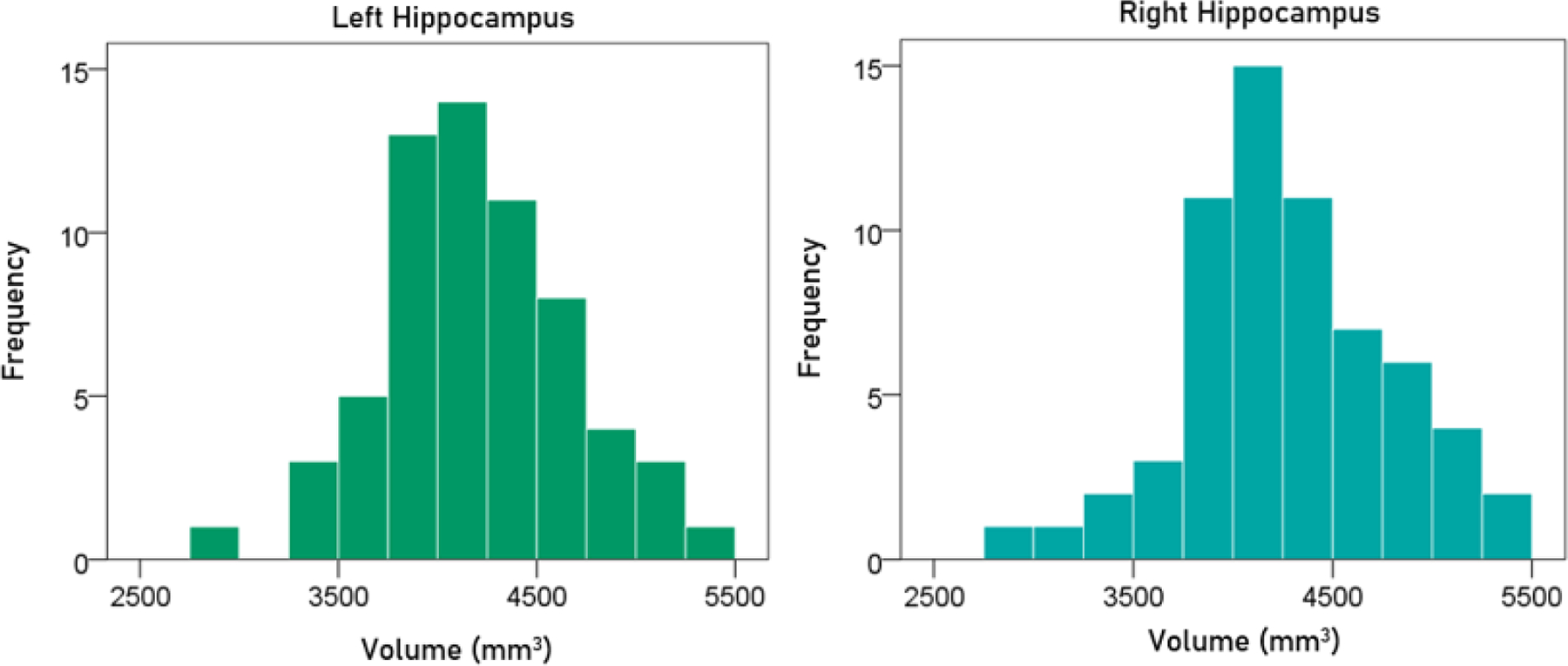
Frequency distribution of the volume of the left and right hippocampus (left and right panels, respectively).

**Figurere 5.**
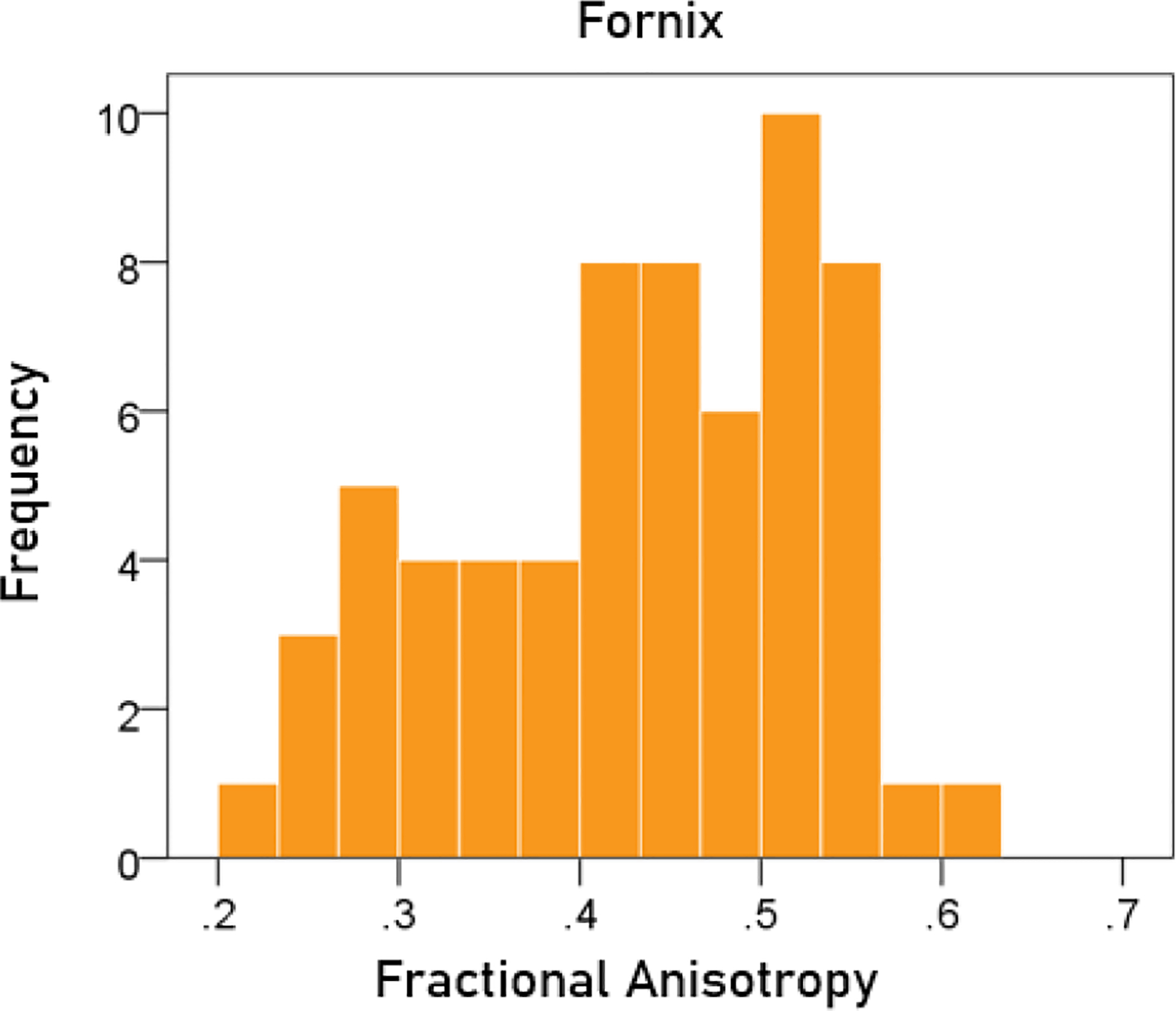
Frequency distribution of fractional anisotropy of the fornix.

**Figurere 6.**
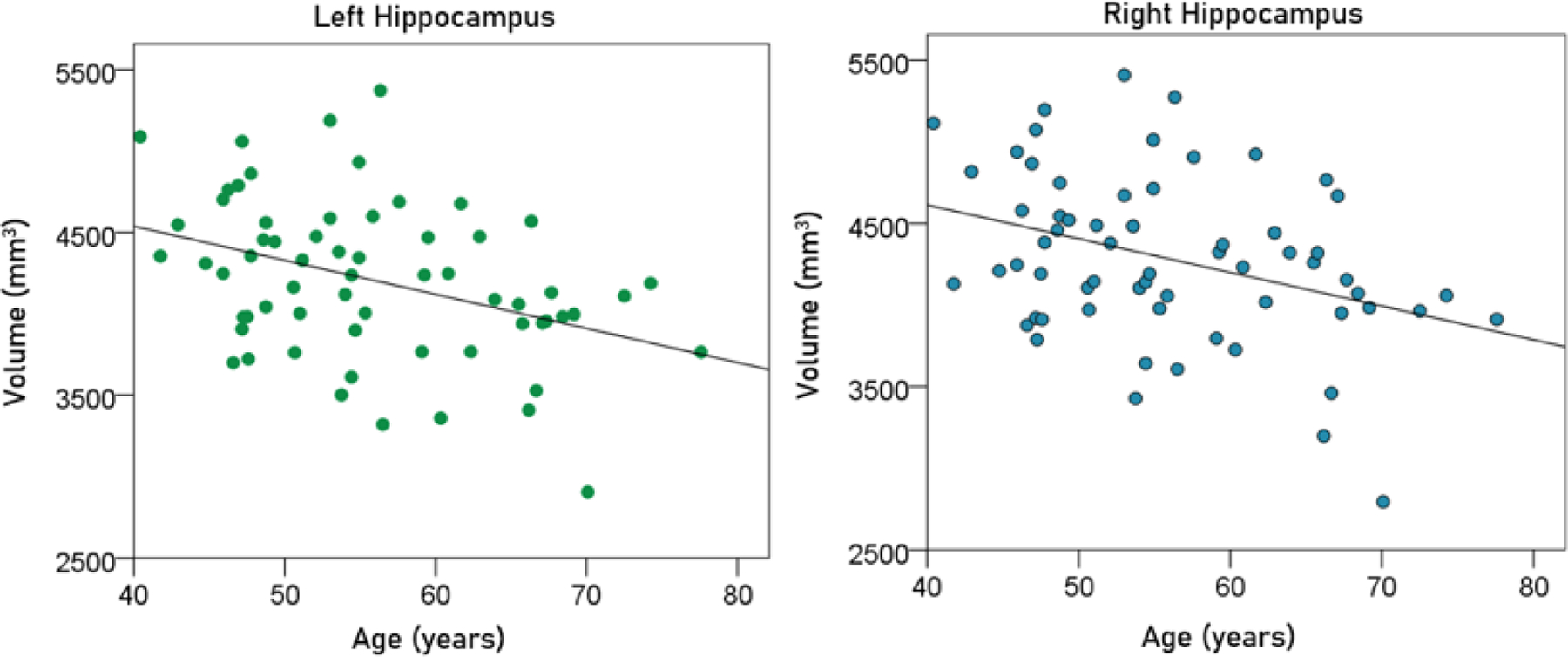
The volumes of left and right hippocampus are plotted against age in the left and right panel, respectively. See text for details.

**Figurere 7.**
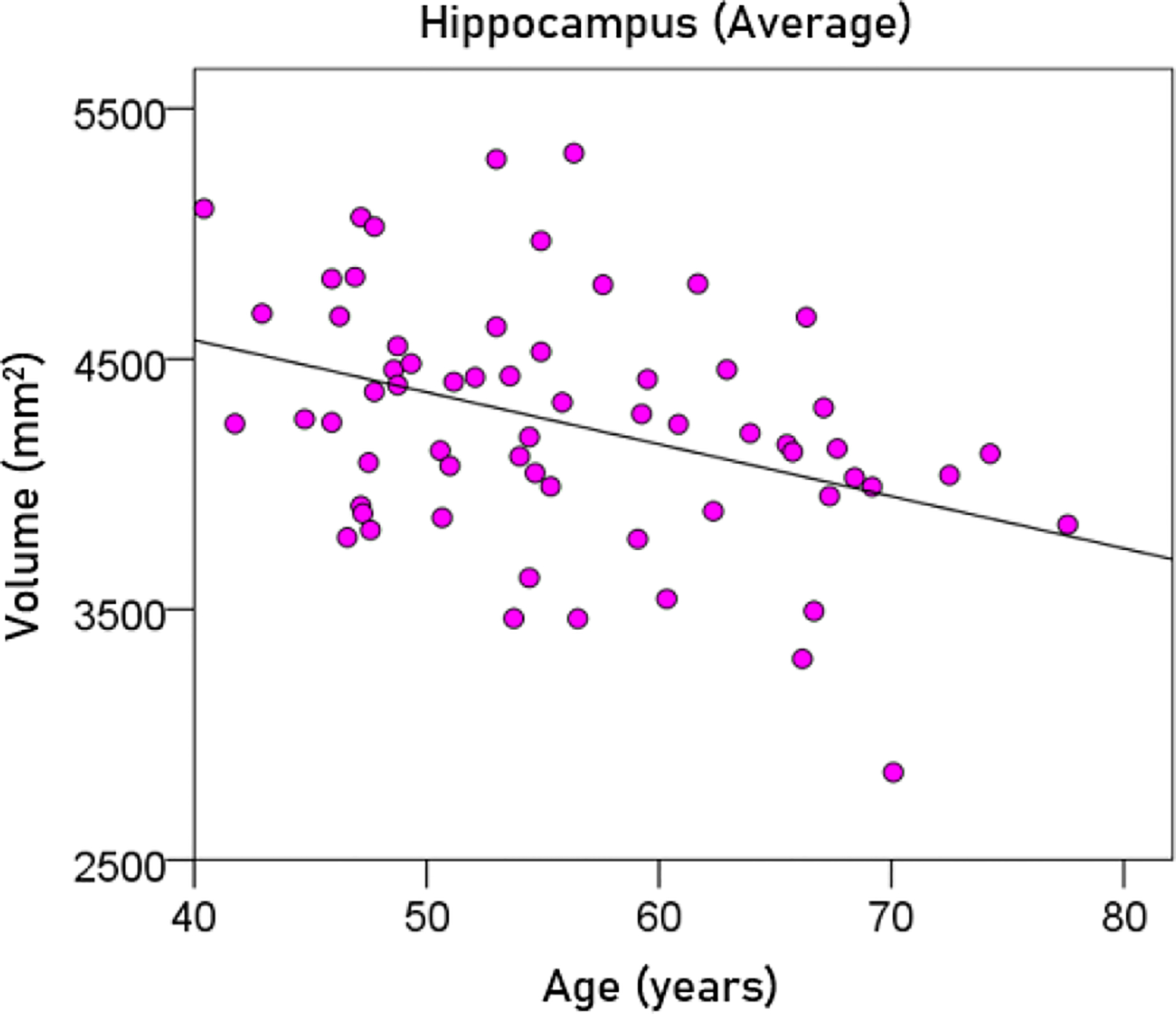
The average hippocampal volume is plotted against age. See text for details.

**Figurere 8.**
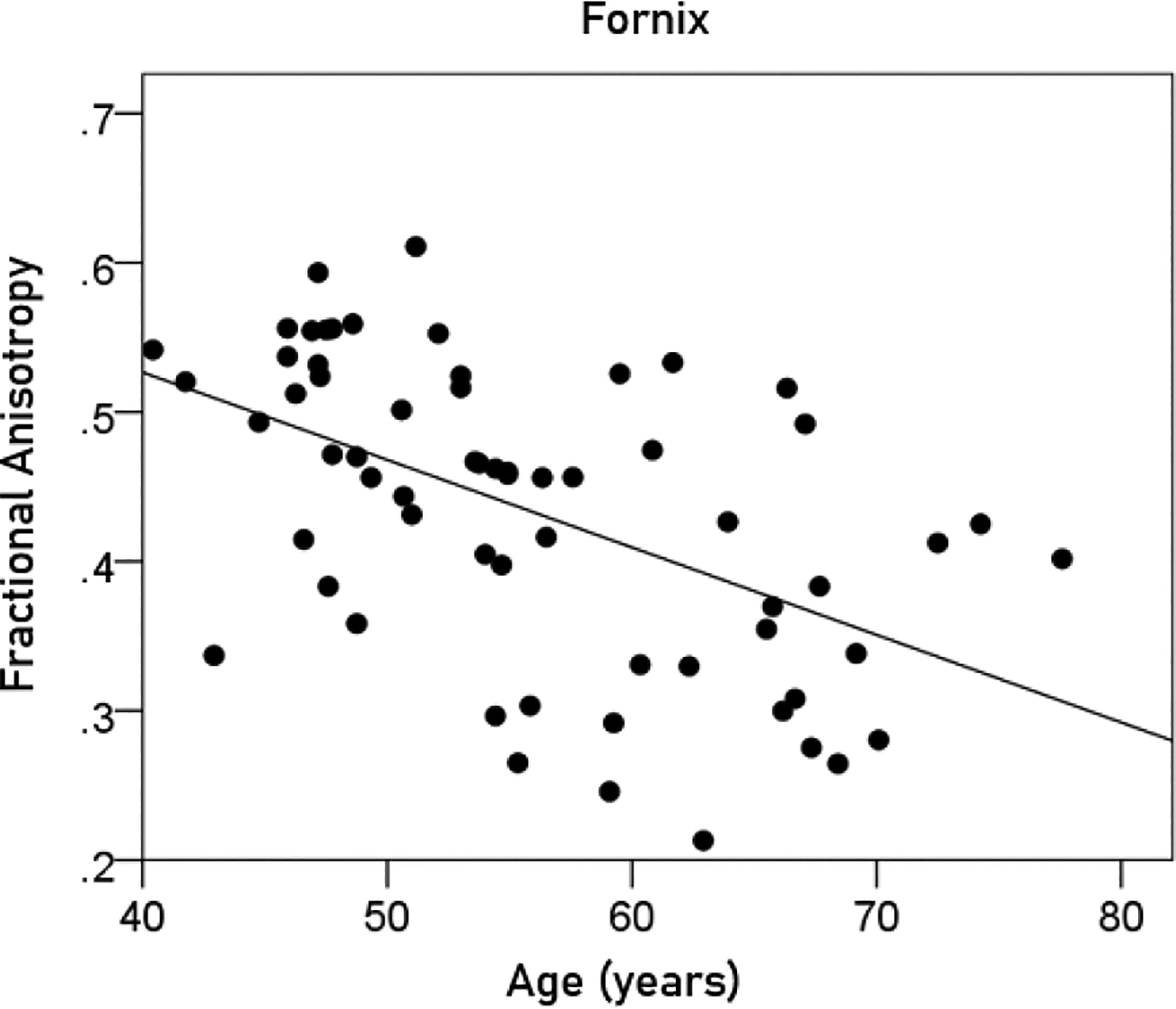
The FA of the fornix is plotted against age. See text for details.

**Figurere 9.**
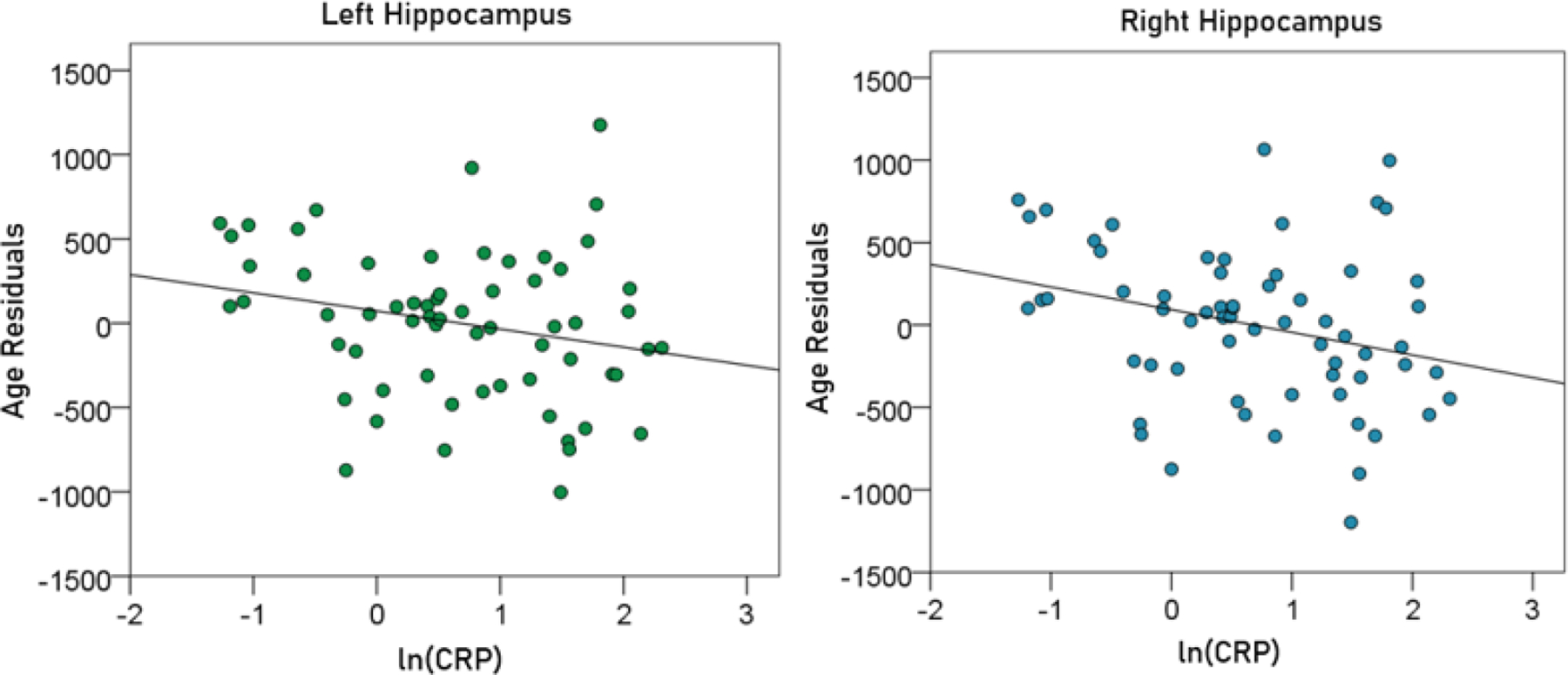
The residual volumes of the left and right hippocampus, after removing the age effect (from Equations 3 and 4, respectively), are plotted against ln(CRP) in the left and right panel, respectively. See text for details.

**Figurere 10.**
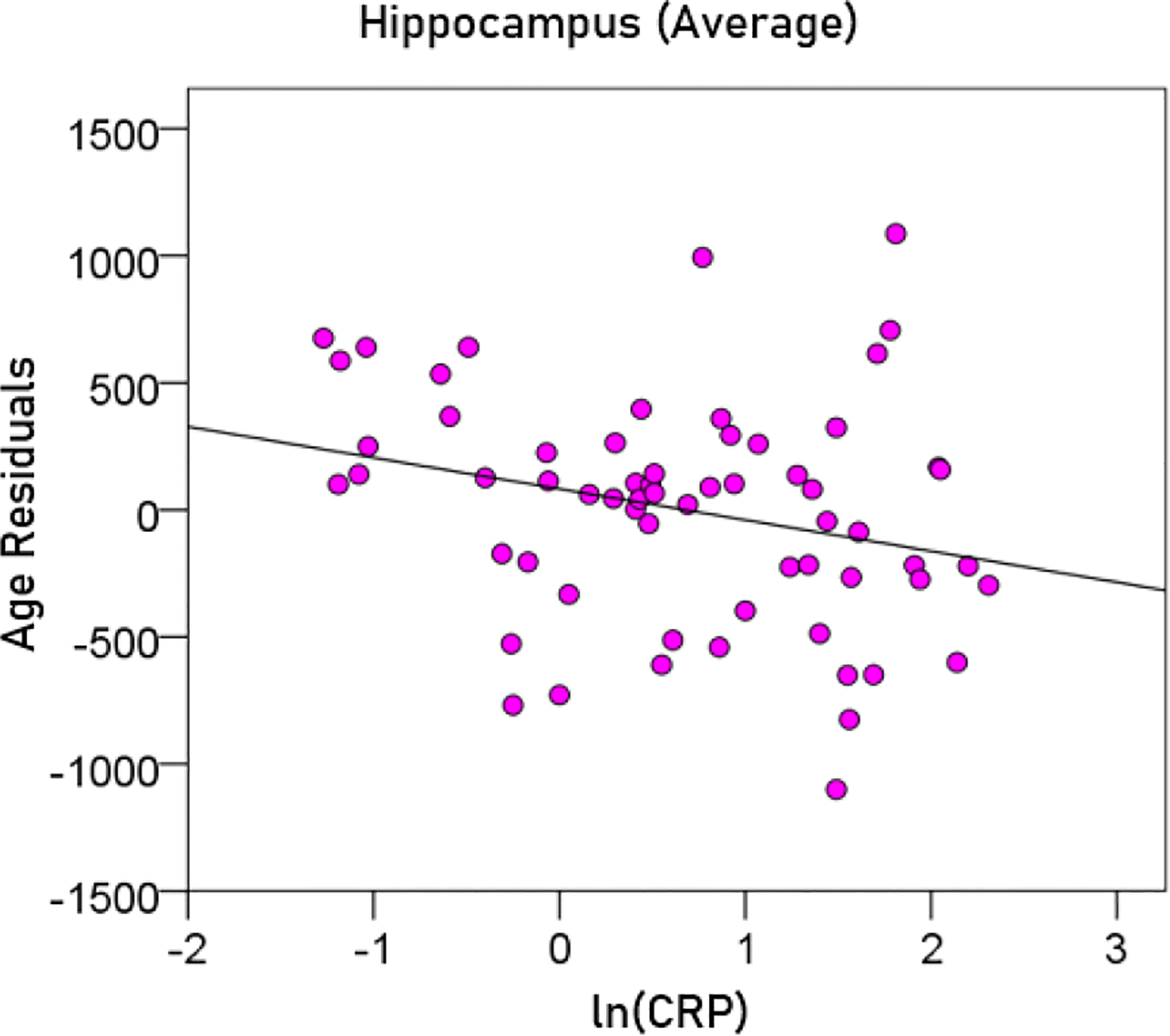
The residual volumes of the averaged hippocampus, after removing the age effect (from Equation 5), are plotted against ln(CRP). See text for details.

**Figurere 11.**
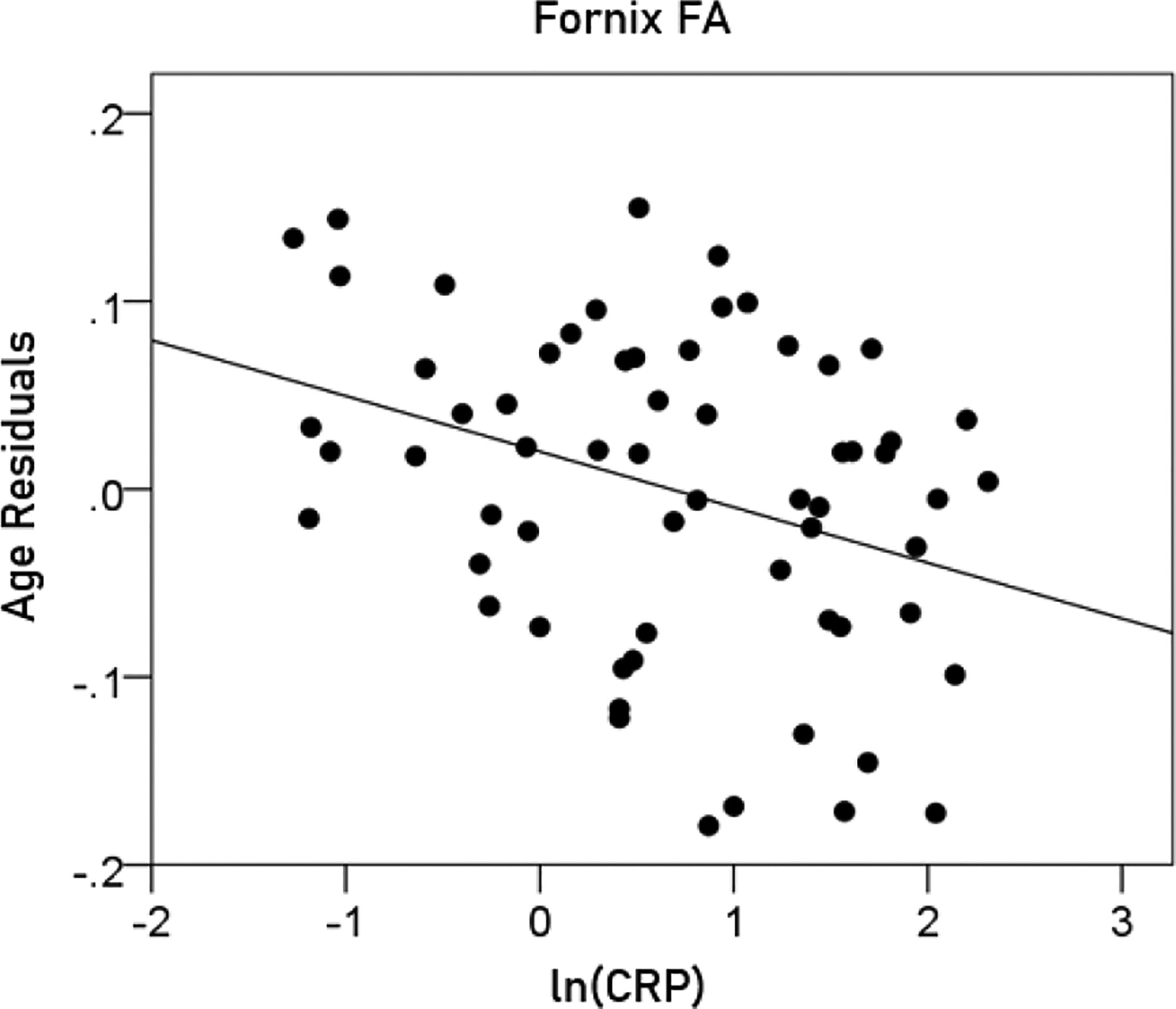
The residual volumes of the fornix FA, after removing the age effect (from Equation 6), are plotted against ln(CRP). See text for details.
